# Determination of *Angptl4* mRNA as a Diagnostic Marker of Primary and Metastatic Clear Cell Renal-Cell Carcinoma

**DOI:** 10.1371/journal.pone.0010421

**Published:** 2010-04-29

**Authors:** Jérôme Verine, Jacqueline Lehmann-Che, Hany Soliman, Jean-Paul Feugeas, Jean-Sébastien Vidal, Pierre Mongiat-Artus, Stéphanie Belhadj, Josette Philippe, Matthieu Lesage, Evelyne Wittmer, Stéphane Chanel, Anne Couvelard, Sophie Ferlicot, Nathalie Rioux-Leclercq, Jean-Michel Vignaud, Anne Janin, Stéphane Germain

**Affiliations:** 1 INSERM, U833, Paris, France; 2 Collège de France, Chaire de Médecine Expérimentale, Paris, France; 3 INSERM, U728, Paris, France; 4 Université Paris Diderot – Paris 7, Paris, France; 5 AP-HP, Hôpital Saint-Louis, Laboratoire de Pathologie, Paris, France; 6 AP-HP, Hôpital Saint-Louis, Laboratoire de Biochimie, Paris, France; 7 INSERM, U944, Paris, France; 8 INSERM, U708, Paris, France; 9 AP-HP, Hôpital Saint-Louis, Service d'Urologie, Paris, France; 10 Laboratoire Pasteur Cerba, Cergy-Pontoise, France; 11 AP-HP, Hôpital Beaujon, Laboratoire de Pathologie, Clichy, France; 12 AP-HP, Hôpital de Bicètre, Laboratoire de Pathologie, Kremlin-Bicètre, France; 13 CHU de Rennes, Laboratoire de Pathologie, Rennes, France; 14 CHU de Nancy, Laboratoire de Pathologie, Nancy, France; University Medical Center Rotterdam, Netherlands

## Abstract

**Background:**

We have previously shown that *angiopoietin-like 4* (*angptl4*) mRNA, a hypoxia-inducible gene, is highly expressed in clear cell renal-cell carcinoma (ccRCC), the most common subtype of RCC for which no specific marker is available. We here investigated whether *angptl4* mRNA 1) could be a useful diagnostic and/or prognostic marker of ccRCC in a large and comprehensive retrospective series, 2) induction is dependent on the *VHL* status of tumors.

**Methodology/Principal Findings:**

Using *in situ* hybridization, we report that *angptl4* mRNA is expressed in 100% of both sporadic (n = 102) and inherited (n = 6) primary ccRCCs, without any statistical association with nuclear grade (*p = 0.39*), tumor size (*p = 0.09*), stage grouping (*p = 0.17*), progression-free survival (*p = 0.94*), and overall survival (*p = 0.80*). *Angptl4* mRNA was also expressed in 26 (87%) of 30 secondary ccRCCs but neither in any other secondary RCCs (n = 7). In contrast, *angptl4* mRNA was neither expressed in 94% non-ccRCC renal tumors (papillary RCCs (n = 46), chromophobe RCCs (n = 28), and oncocytomas (n = 9)), nor in non-renal clear cell carcinomas (n = 39). *Angptl4* expression was also examined in tumors associated (n = 23) or not associated (n = 66) with VHL disease. 40 (98%) hemangioblastomas expressed *angptl4* whereas all pheochromocytomas (n = 23) and pancreatic tumors (n = 25) were *angptl4*-negative, whatever their *VHL* status.

**Conclusions/Significance:**

A*ngptl4* mRNA expression was highly associated with ccRCC (*p* = 1.5 10^−49^, Chi square test) allowing to define its expression as a diagnosis marker for primary ccRCC. Moreover, *angptl4* mRNA allows to discriminate the renal origin of metastases of clear-cell carcinomas arising from various organs. Finally, inactivation of *VHL* gene is neither necessary nor sufficient for *angptl4* mRNA induction.

## Introduction

Renal-cell carcinoma (RCC) represents 2% of all malignant diseases in adults and is the third most common genitourinary cancer site, after prostate carcinoma and transitional cell carcinoma of the urinary bladder [1]. RCC is estimated to account for 2.3% of all cancer-related deaths; this corresponds to ∼26,400 patients in Europe [2] and ∼102,000 in the world [3], [4]. The histological subtypes of RCC are numerous, principally represented by clear cell RCC (ccRCC), papillary RCC (pRCC), chromophobe RCC (chRCC), and carcinoma of the collecting ducts. Moreover, several benign renal tumors are also described, including oncocytoma, papillary adenoma, and angiomyolipoma [5].

Clear cell RCC (ccRCC), the most common subtype of RCC, represents more than 75% of all cases [5], [6]. An early event during the evolution of ccRCC is loss of function of the von Hippel-Lindau (*VHL*) gene, a tumor suppressor gene located on 3p25.3 [6]. The best documented function of the *VHL* gene product, pVHL, relates to its role as the substrate recognition module of an ubiquitin ligase complex that targets the α-subunit of hypoxia-inducible factor (HIF) for destruction in the proteasome. Through transcriptional regulation, HIF enhances glucose uptake and increases expression of angiogenic, growth, and mitogenic factors including vascular endothelial growth factor (VEGF), platelet derived growth factor β polypeptide (PDGFβ), erythropoietin, and transforming growth factor α (TGFα) [7], [8]. Several HIF-independent functions of pVHL have also been described [7], [9]. Germline mutations of the *VHL* gene are responsible for the VHL disease, an autosomal dominant hereditary disorder characterized by the development of benign and/or malignant tumors in different organs. VHL-associated tumors include central nervous system hemangioblastoma, retinal angioma, pheochromocytoma, pancreatic endocrine and serous tumors, endolymphatic sac tumor, papillary cystadenoma of epididymis and broad ligament, and renal cysts and multifocal or bilateral ccRCC; which is the major cause of death of VHL patients [8].

Most renal tumors can be diagnosed by experienced pathologists on the basis of hematoxylin and eosin (H&E) morphology alone. Nevertheless, a morphologic overlap exists between the different histological subtypes of RCC, and sometimes renal oncocytoma and epithelioid angiomyolipoma enter into the differential diagnosis. Since RCC subtypes have different malignant potential, prognoses and optimal therapies [10], many markers have been tested to support an accurate histological classification, including CD10, RCC marker (RCCma), CK7, CD117, CA9 and AMACR. Unfortunately, none of these markers is to date specific for one subtype of renal epithelial tumor [10], [11], [12], [13]. Different panels of markers for differential diagnosis of renal epithelial tumors have also been proposed [14], [15]. Nevertheless, they are heterogeneous in number and nature of markers, and so far they have a limited application for daily practice.

Besides, the improvement of imaging techniques for small tumors detection and the increasing use of minimally invasive destructive technology (ie, radiofrequency and cryotherapy) require imaging-guided renal biopsy to analyse these renal tumors. The need of sensitive and specific diagnostic markers becomes even more evident in this context.

Angiopoietin-like 4 (ANGPTL4), initially known as hepatic fibrinogen/angiopoietin-related protein (HFARP), peroxisome proliferator-activated receptor-γ (PPARγ) angiopoietin-related gene (PGAR), or fasting-induced adipose factor (FIAF), is a secreted glycoprotein which structurally belongs to the angiopoietin/ANGPTL family [16]. This protein family comprises at least 11 members with a molecular weight of 44 to 58 kDa. Human ANGPTL4 consists of 406 amino acids protein with a signal peptide directing secretion, an amino-terminal coiled-coil domain, a linker, and a carboxy-terminal fibrinogen-like domain [17], [18]. The most studied function of ANGPTL4 is its role in the regulation of lipid metabolism, particularly as inhibitor of lipoprotein lipase activity [18]. Its role in vascular and tumor processes is more debated [16], [17], [19], and suggestive of a context, tissue specific activity of ANGPTL4.

Our laboratory has previously shown that *ANGPTL4* is a hypoxia-inducible gene. Indeed, *angptl4* mRNA is expressed in ischemic tissues and in the perinecrotic areas of different human tumors. Interestingly, it is also highly up-regulated in ccRCC [20].

The present study was designed first, to determine whether *angptl4* mRNA expression is a useful marker of ccRCC in a large and comprehensive retrospective series of primary and metastatic renal tumors, including the uncommon subtypes; second, to evaluate whether *angptl4* mRNA expression is able to determine the renal origin of metastatic clear cell carcinomas by analysing *angptl4* mRNA expression in non-renal clear cell carcinomas; third, to determine whether *angptl4* mRNA expression has any prognostic value in ccRCC; and fourth, to define whether the induction of *angptl4* mRNA expression in human tumors is dependent of VHL pathway by examining both the *VHL* status of a subgroup of *angptl4*- positive RCC and *angptl4* mRNA expression in the main non-renal VHL disease-related tumors. Our study demonstrates that *angptl4* mRNA expression is a diagnostic marker of primary and metastatic ccRCC but has no prognosis value. Moreover, inactivation of *VHL* gene is neither necessary nor sufficient for *angptl4* mRNA induction.

## Methods

### Patients and tumor samples

#### Renal tumors

All formalin-fixed, paraffin-embedded samples of primary renal tumors obtained from patients who underwent radical or partial nephrectomy at Hôpital Saint-Louis (Paris, France) during the 2002–2006 period were gathered for this study. The inclusion of uncommon subtypes of renal tumors was extended to the 2000–2008 period. Finally, renal pediatric tumors and 5 cases of VHL disease-associated ccRCC with a documented germline mutation were retrieved from the files of the departments of Pathology of Hôpital Pontchaillou (Rennes, France), and Hôpital de Bicêtre (Kremlin-Bicêtre, France), respectively. One pathologist (JV) reviewed the H&E sections of all primary renal tumors (n = 253) and determined the histopathological diagnosis according to the current 2004 World Health Organization classification [5]. These tumors included 108 ccRCCs (42.7%, sporadic ccRCCs [n = 102], ccRCCs associated with VHL disease [n = 5] or tuberous sclerosis complex (TSC) [n = 1]), 3 multilocular cystic RCCs (1.2%), 46 pRCCs (18.2%, type 1 [n = 32], type 2 [n = 10], and oncocytic variant [n = 4]), 3 mucinous tubular and spindle cell carcinomas (1.2%), 28 chRCCs (11%), 2 hybrid oncocytic tumors in Birt-Hogg-Dube (BHD) syndrome (0.8%), 2 carcinomas of the collecting ducts (0.8%), 1 medullary carcinoma (0.4%), 2 Xp11.2 translocation carcinomas (0.8%), 6 nephroblastomas (Wilm's tumors) (2.4%), 2 clear cell sarcomas (0.8%), 4 urothelial carcinomas of the renal pelvis (1.6%), 9 oncocytomas (3.5%), 12 angiomyolipomas (4.7%), 11 papillary adenomas (4.3%), 3 metanephric adenomas (1.2%), 1 cystic nephroma (0.4%), 4 renomedullary interstitial cell tumors (1.6%) and 1 mixed epithelial and stromal tumor (0.4%). Five clear cell papillary RCCs (ccpRCCs; 2%), a new entity recently described [5], were finally included in the study. The nuclear grade was determined using the criteria of Fuhrman [21]. Renal cancers were staged according to the International Union Against Cancer 2002 TNM classification [22]. The clinical data and follow-up of patients with sporadic ccRCC were collected from the clinical records of patients.

Thirty-four samples of RCC in metastatic location (28 ccRCCs; 4 pRCCs, type 1 [n = 1], type 2 [n = 3]; 1 chRCC; 1 medullary) and 3 local recurrences (ccRCCs [n = 2], chRCC [n = 1]) were also gathered for this study. Among them, 20 occurred in patients whose primary tumor was included in the present study, and 17 came from biopsy or resection of metastatic tumors only. Finally, the renal parenchyma adjacent to the tumor was used as a source of normal renal tissue.

#### Non-renal VHL disease-related tumors

Forty-one hemangioblastomas and 25 pancreatic tumors (19 endocrine and 6 serous tumors) retrieved from the archives of the Departments of Pathology of CHU de Nancy (Nancy, France), and Hôpital Beaujon (Clichy, France) were collected for this study. Among these tumors, 5 hemangioblastomas, and 13 pancreatic tumors (13 endocrine and 2 serous tumors) were VHL disease-associated tumors with a documented germline mutation in the *VHL* gene. All hemangioblastomas and VHL disease-associated pancreatic tumors included in this study were previously reported in 2 distinct studies [23], [24].

In addition, 23 apparently sporadic pheochromocytomas, carrying no evidence of underlying inherited tumor syndromes, including multiple endocrine neoplasia type 2, VHL disease, neurofibromatosis type 1, and the pheochromocytoma–paraganglioma syndrome, were provided by the Pathology department of Hôpital Saint-Louis (Paris, France).

#### Non-renal clear cell carcinomas

Twelve ovarian clear cell adenocarcinomas, 20 endrometrial clear cell adenocarcinomas, and 7 clear cell variants of lung carcinoma (adenocarcinomas [n = 5] and epidermoid carcinomas [n = 2]) were obtained from the Departments of Pathology of Pasteur Cerba (Cergy-Pontoise, France), and CHU de Nancy (Nancy, France).

The study was approved by the Institutional Review Board (Hôpital Saint-Louis, Paris, France). Written informed consent was obtained for the study according to the National Institute of Cancer professional recommendations and the French Law of August 2004 (loi 2004-800 du 6 août 2004) and according to the Declaration of Helsinki Principles.

### Procedures

#### *In situ* hybridization

5-µm tissue sections were mounted on superfrost plus slides. The detailed protocol for *in situ* hybridization has been previously published [20]. Observation was performed under a microscope with dark field or bright field illumination. The *angptl4* mRNA expression level was evaluated in a blinded fashion using sense probe as a negative control. *in situ* hybridization allows to determine *angptl4* mRNA-positive cell-types (ie, tumor or stromal cells). The extent and the intensity of signal were evaluated using a semiquantitative scoring method as: 0 = absent; 1 = mild and focal; 2 = moderate and focal; 3 = strong and focal; and 4 = strong and diffuse, in comparison to the same ccRCC specimen (score 3) which was systematically included in each hybridization series and was therefore used as a reference in each hybridization series.

#### VHL gene status analyses

*VHL* gene status analyses were performed without knowledge of any pathological or clinical data in 2 independent experiments. For each case, ten 7-µm sections of formalin-fixed paraffin-embedded tissue blocks were obtained from paired tumor and normal tissue specimens containing >90% of tumor cells and no tumor cells, respectively. DNA was extracted using the QIAamp DNA Mini Kit (Qiagen, Courtaboeuf, France) according to the manufacturer instruction.

For *VHL* gene mutation analysis, PCR based amplification of each of the 3 coding exons was performed on a Lightcycler 2.0 System (Roche) using SyberGreen Master Mix (Roche). Two hundred ng genomic DNA were amplified according to conditions and using primers listed in Table S1. Amplicons were then purified and sequenced on both strands using the BigDye Terminator v3.1 kit (Applied Biosystems, conditions: 96°C, 5mn, 1 cycle, 96°C, 10 sec, 60°C 5 sec, 60°C 4mn, 25 cycles) and analysed on a 3130 Genetic Analyser (Applied Biosystems). The resulting sequences were compared to that of 3 *VHL* exons using the SeqScape software (Applied Biosystems). The identified mutations were compared to mutations described in the *VHL* database (http://www.umd.be/VHL/).

For microsatellite analysis, allelotyping of a physical distance of approximately 6.5 Mb between markers D3S1560 and D3S3611 was performed using 6 polymorphic microsatellite markers flanking *VHL* gene and listed in the UCSC database in the order telomeric to centromeric 3p: D3S1560 (3p26.2), D3S1597 (3p25.3), D3S1317 (3p25.3), D3S1435 (3p25.3), D3S1038 (3p25.3), and D3S3611 (3p25.3). *VHL* gene is located between D3S1597 and D3S1317. The primer sequences used for PCR analyses are listed in Table S2. One primer of each microsatellite marker was end-labelled with one of the following fluorophores: 6-FAM, NED® or HEX (Applied Biosystems). PCR was conducted as follows: for each microsatellite marker, equal amounts of DNA (20 ng each of constitutional and tumor-extracted) were subjected simultaneously in 2 different PCR tubes to one cycle of soaking at 95°C in a GeneAmp PCR System 9700 (Applied Biosystems), then to 40 cycles of amplification at 95°C for 30 sec and 60°C for 1 min. The amplification was terminated by a final extension step at 72°C for 10 min. The resulting PCR products were analysed on an Applied Biosystems 3130xl genetic analyser using the GeneMapper software. This technique allowed the estimation of allele size and the quantitative evaluation of allele ratio. Homozygous markers were quoted “not informative”. Informative cases were scored as LOH when the intensity of the signal for one allele in tumor tissue specimens was decreased by >50% in comparison to allelic signal observed in normal tissue specimens. Allelic imbalance was scored when the signal for one allele was decreased >20% and <50%.

### Statistical analysis

Analyses were performed using R packages (The R Project for Statistical Computing, version 2.10.1). The performance of *angptl4* mRNA expression for diagnostic of ccRCC was assessed by considering the number of correctly classified (true positives, TP, and true negatives, TN) and incorrectly classified (false positives, FP, and false negatives, FN) cases, and a Chi-square test was performed. The correlation studies between *angptl4* mRNA expression level and the main histological prognostic factors of ccRCC (ie, Fuhrman nuclear grade, 2002 AJCC TNM stage groupings, and tumor size), between *angptl4* mRNA expression level and *VHL* mutations were statistically analysed using the Fisher's exact test for qualitative variables and the Kruskal-Wallis K test for quantitative variables (R-package “stats”). Progression–free survival (PFS) and overall survival of patients with ccRCC were estimated by the Kaplan-Meier method (R-package “survival”). PFS was defined as the time from start of therapy to the occurrence of local recurrence or distant metastases. Overall survival was defined as the time from start of therapy to death due to any cause. For each test, only p values lower than 0.05 were considered statistically significant.

## Results

### *Angptl4* mRNA is a diagnostic marker of primary and secondary ccRCCs

**Figure 1:**
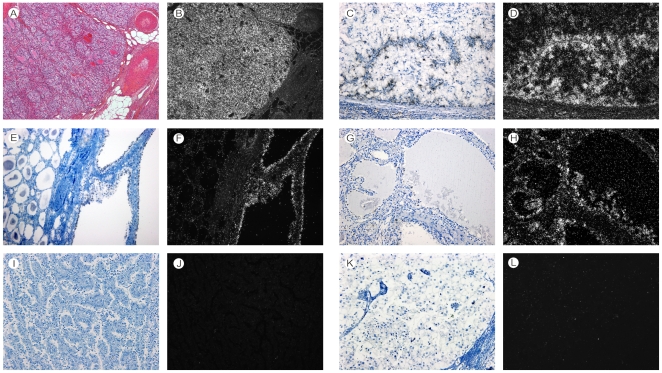
In situ hybridization analysis of *angptl4* mRNA expression in renal tumors. H&E (A) and dark-field (B) views of *angptl4* mRNA expression in ccRCC (×4). Bright-field (C) and dark-field (D) views of lymph node metastasis of ccRCC (×10). Bright-field (E) and dark-field (F) views of *angptl4* mRNA expression in multilocular cystic RCC (×10). Bright-field (G) and dark-field (H) views of *angptl4* mRNA expression in ccpRCC (×10). Bright-field (I) and dark-field (J) views of *angptl4* mRNA expression in type 2 pRCC (×10). Bright-field (K) and dark-field (L) views of *angptl4* mRNA expression in chRCC (×10). Note: Strong expression of *angptl4* mRNA in primary (A, B) and secondary ccRCC (C, D), in multilocular cystic RCC (E, F), and in ccpRCC (G, H). Absence of *angptl4* mRNA expression in type 2 pRCC (I, J), and chRCC (K, L).

*Angiopoietin-like 4* mRNA expression was assessed in 253 primary renal tumors using *in situ* hybridization which allowed us to determine both expression level and the cell types that express it. Heteregenous expression of *angptl4* mRNA was detected in all 108 cases (100%) of ccRCCs, independently of their sporadic or inherited (VHL or TSC diseases) origin (Figure 1A, B; Table 1). Expression was systematically detected in tumor cells but not in stromal cells (ie, endothelial and inflammatory cells, myocytes, fibroblasts and myofibroblasts). *Angptl4* mRNA expression was also detected in 26 cases (86.7%) of 30 metastases or local recurrence of ccRCC, as shown in Figure 1C, D. The primary tumor was available in 14 of these 30 secondary ccRCCs. By comparison to the primary tumor, the expression level in secondary tumor was similar in 10 cases (71.4%), weaker in 1 case (7.1%), and absent in 3 cases (21.5%).

**Table 1:** *angptl4* mRNA expression in 41 benign and 212 malignant renal tumors.

Renal tumors	Number of tumors	*angptl4* mRNA-positive tumors	*angptl4* mRNA-negative tumors
**Malignant tumors (n = 212)**			
Clear cell Renal Cell Carcinoma (ccRCC)	108	108 (100%)	0
Sporadic ccRCC	102	102 (100%)	0
VHL-associated ccRCC	5	5 (100%)	0
Tuberous sclerosis complex-associated ccRCC	1	1 (100%)	0
Multilocular cystic RCC	3	3 (100%)	0
Clear cell papillary RCC	5	5 (100%)	0
Papillary RCC	46	0	46 (100%)
Mucinous tubular and spindle cell carcinoma	3	0	3 (100%)
Chromophobe RCC	28	0	28 (100%)
Hybrid oncocytic tumor (BHD)	2	0	2 (100%)
Collecting duct carcinoma	2	0	2 (100%)
Medullary carcinoma	1	0	1 (100%)
Xp11⋅2 translocation carcinoma	2	0	2 (100%)
Nephroblastoma (Wilm's tumor)	6	1 (17%)*	5 (83%)
Clear cell sarcoma	2	0	2 (100%)
Urothelial carcinoma of renal pelvis	4	0	4 (100%)
**Benign tumors (n = 41)**			
Oncocytoma	9	0	9 (100%)
Angiomyolipoma	12	0	12 (100%)
Papillary adenoma	11	0	11 (100%)
Metanephric adenoma	3	0	3 (100%)
Cystic nephroma	1	0	1 (100%)
Renomedullary interstitial cell tumor	4	0	4 (100%)
Mixed epithelial and stromal tumor	1	0	1 (100%)

*angptl4 mRNA expression is limited to a small and cystic area lined by clear cells.

In addition to ccRCC, *angptl4* mRNA was detected in all other clear cell renal epithelial tumors (n = 8) included in the present study. Indeed, all 3 cases (100%) of cystic multilocular RCC (figure 1E, F), and all 5 cases (100%) of ccpRCC (figure 1G, H) expressed *angptl4* mRNA. In addition, 1 nephroblastoma also expressed *angptl4* mRNA in a focal area of small cysts lined by clear cells, whereas the rest of the tumor which devoid of clear cells was negative.

In contrast, *angptl4* mRNA was never expressed in the other benign (n = 41) and malignant (n = 95) primary renal tumors, including pRCCs (figure 1I, J), chRCCs (figure 1K, L), and oncocytomas. All 7 secondary non-clear cell RCCs (2 chRCCs, 4 pRCCs, 1 medullary carcinoma) were also negative for *angptl4* mRNA expression.

Besides, as previously described [20], *angptl4* mRNA expression was detected in the perinecrotic region of several types of primary renal tumors, represented essentially by pRCCs and ccRCCs, and in some metastases of RCC. This mRNA expression pattern was not specific of a subtype of renal tumors but depended on the existence or not of necrotic areas in tumors. These *angptl4*-positive perinecrotic areas were very limited and not considered in the determination of *angptl4*-positive or negative tumors.

Finally, normal renal tissue adjacent to the tumor rarely expressed *angptl4* mRNA (7.4% of cases), in some distal and collector tubules.

In summary, in this large and comprehensive series, *angptl4* mRNA expression was expressed in 100% of ccRCCs and in only 6.8% of non-ccRCC renal tumors. Thus, *angptl4* mRNA expression was highly associated with ccRCC (*p* = 1.5 10^−49^, Chi square test). The *angptl4* mRNA expression was also limited to ccRCC subtype in local recurrence and metastatic cases of RCC (ie, 86.7% [26 of 30] positive in secondary ccRCCs, and 0% [0 of 7] positive in non-ccRCC secondary renal tumors).

Since differential diagnosis of metastatic clear cell carcinomas is often difficult, we also analysed *angptl4* mRNA expression in 12 cases of ovarian clear cell adenocarcinoma, 20 cases of endometrial clear cell adenocarcinoma, and 7 cases of clear cell variants of lung carcinoma. None of these tumors expressed *angptl4* mRNA (Table 2).

**Table 2:** *angptl4* mRNA expression in 89 VHL disease-associated tumors and 39 non-renal clear cell carcinomas.

Tumor types	Number of tumors	*angptl4* mRNA-positive tumors	*angptl4* mRNA-negative tumors
**VHL disease-associated tumor (n = 89)**			
Hemangioblastoma (n = 41)			
Sporadic hemangioblastoma	36	36 (100%)	0
VHL disease-associated hemangioblastoma	5	4 (80%)	1 (20%)
Pancreatic tumor			
Endocrine tumor (ET) (n = 19)			
Non VHL disease-associated ET	6	0	6 (100%)
VHL disease-associated ET	13	0	13 (100%)
Serous tumor (ST) (n = 6)			
Non VHL disease-associated ST	4	0	4 (100%)
VHL disease-associated ST	2	0	2 (100%)
Pheochromocytoma (PCC) (n = 23)			
Non VHL disease-associated PCC	20	0	20 (100%)
VHL disease-associated PCC	3	0	3 (100%)
**Non-renal clear cell carcinoma (n = 39)**			
Ovarian clear cell adenocarcinoma	12	0	12 (100%)
Endometrial clear cell adenocarcinoma	20	0	20 (100%)
Clear cell adenocarcinoma of lung	5	0	5 (100%)
Clear cell epidermoid carcinoma of lung	2	0	2 (100%)

### *Angptl4* mRNA has no prognostic value in ccRCCs

In order to determine whether *angptl4* mRNA level had a prognostic value in ccRCC, a correlation study was performed in all sporadic ccRCC cases (n = 102), independently of clinical data and follow-up of the patients. The levels of *angptl4* mRNA expression were not significantly different between low (G1/G2) vs. high (G3/G4) Fuhrman nuclear grade (*p = 0.39*, see Table 3). Also, *angptl4* mRNA expression level was not statistically associated with tumor size (*p = 0.09*), and 2002 AJCC TNM stage grouping (*p = 0.17*). The follow-up of patients with ccRCC ranged from 1 to 99 months after tumor surgical resection, with a mean (standard deviation) of 36.2 (25.5) months (see Table 3). In this study, the estimate probabilities of both local recurrence or distant metastases, and death by five years was 0.35 (95% CI 0.22 to 0.46), and 0.21 (95% CI 0.10 to 0.31), respectively. The outcome of these patients showed no difference in the occurrence of local recurrence and/or metastases according to *angptl4* mRNA expression level (score 1 vs. score 2–3 vs. score 4; *p = 0.97*). No difference was also observed in PFS (*p = 0.94*), and overall survival (*p = 0.80*).

**Table 3:** Correlation study between *angptl4* mRNA expression and prognostic factors and *VHL* status in sporadic ccRCC patients.

	Intensity of *angptl4* mRNA expression			
	Score 1	Score 2–3	Score 4	
	(n = 11)	(n = 75)	(n = 16)	*p*-Value†
**Nuclear grade**				0.39
G1–G2	3 (7%)	34 (81%)	5 (12%)	
G3–G4	8 (14%)	41 (68%)	11 (18%)	
**TNM Stage grouping**				0.17
I–II	4 (6%)	49 (79%)	9 (15%)	
III–IV	7 (17%)	26 (65%)	7 (18%)	
**Tumor size (cm) M (SD)** *	4.7 (2.3)	5.9 (3.6)	7.1 (3.4)	0.09¶
**Follow-up M (SD)** *	22 (17.5)	37.1 (27)	39.9 (22)	0.18¶
**Metastasis and/or local recurrence**				0.97
No	8 (11%)	53 (73%)	12 (16%)	
Yes	3 (10%)	22 (76%)	4 (14%)	
***VHL*** ** gene status**				0.01
Mutation	0	12 (57%)	9 (43%)	
Wild type	7 (28%)	14 (56%)	4 (16%)	

*M (SD): mean (standard deviation).

†Fisher exact Test.

¶Kruskall-Wallis Test.

### *Angptl4* mRNA induction is independent of *VHL* gene inactivation

The contribution of *VHL* alterations in *angptl4* mRNA expression was assessed in human tumors by analysis of the *VHL* status in 51 (50%) of the 102 sporadic ccRCCs. Sequencing analysis of the 3 coding exons of the *VHL* gene and a search for an allelic imbalance or LOH in the *VHL* gene locus were performed on paraffin-embedded or frozen tissue specimens. Forty-six cases (90.2%) were available for sequencing analysis. A *VHL* gene intragenic mutation was noted in 21 cases (45.6%), including 1 case with a biallelic mutation (case n°38, Table 4). The majority (63.6%) of these mutations corresponded to single-nucleotide substitutions (missense and nonsense mutations).

**Table 4:** Somatic mutations of the *VHL* gene in a subgroup of ccRCCs (n = 46).

Tumor number	Type of mutation	Exon	Coding DNA sequence change	Protein change (deduced)	Previously reported
12	Missense	1	c.233A>T	p.Asn78IIe	Yes
18	Missense	1	c.257C>T	p.Pro86Leu	Yes
21	Missense	1	c.234T>G	p.Asn78Lys	Yes
25	Missense	3	c.473T>G	p.Leu158Arg	No
26	Frameshift	1	c.192delC	p.Ser65ArgfsX2	Yes
32	Frameshift	2	c.345delC	p.Leu116PhefsX43	Yes
33	Large rearrangement	−	−	−	−
38	Missense and nonsense	1	c.[262T>G + 263 G>A]	p.[Trp88Glu + Trp88X]	Yes
40	Missense	2	c.397C>G	p.Thr133Ser	Yes
42	Frameshift	2	c.388delGTT	p.Val130del	Yes
45	Nonsense	1	c.203 C>A	p.Ser68X	Yes
52	Missense	1	c.241C>T	p.Pro81Ser	Yes
53	Missense	2	c.367G>T	p.Thr123Leu	No
62	Frameshift	1	c.322–324delCGC	p.Arg107del	No
63	Missense	3	c.617T>C	p.Ile206Thr	No
64	Missense	2	c.446C>A	p.Ala149Asp	No
67	Missense	1	c.206G>A	p.Arg69His	No
73	Nonsense	3	c.478G>T	p.Glu160X	Yes
75	Frameshift	3	c.477delA	p.Glu160SerfsX10	Yes
77	Frameshift	1	c.338–349del	p.Arg113del+Splice defect	No
99	Frameshift	1	c.256delC	p.Pro86ProfsX	Yes

Mutations are described using ‘p.’ when referring to the VHL protein sequence, and ‘c.’ for the *VHL* cDNA sequence. Mutations are reported in accordance with the nomenclature for the description of sequence variations as proposed by the Human Genome Variation Society (www.hgvs.org/mutnomen/).

The result of LOH at the *VHL* gene locus is shown in Table 5. Fifty cases were available for LOH analysis. Six cases (12%) were not informative (cases n°1, 27, 42, 57, 60, and 68, Table 5). Two cases (n°53 and 99, Table 5), with homozygous markers in normal tissue specimen, presented one allele with a deletion of some base-pairs in tumor tissue specimen and were considered as informative cases with allelic mutation. Thus, 43 (97.7%) of the 44 informative ccRCCs showed a LOH, an allelic imbalance, or an allelic mutation at the *VHL* gene locus. Among them, 19 tumors (44.2%) presented a *VHL* mutation detected by sequencing. Moreover, a *VHL* gene mutation was noted in 1 of the 6 non informative ccRCCs (case n° 42, Table 4), and in case n°52, not available for LOH analysis because of the absence of normal tissue specimen. Finally, 45 cases (97.8%) out of 46 ccRCCs had a detectable *VHL* mutation and/or a LOH at the *VHL* gene locus. In one case of informative ccRCC, neither *VHL* mutation nor LOH at the *VHL* gene locus were identified (case n°20, Table 5). This unique case was also tested for hypermethylation of the promoter region of the *VHL* gene and was negative (data not shown). Nevertheless, it expressed high lvels of *angptl4* mRNA (score 3).

**Table 5:** Results of LOH analysis at the *VHL* gene locus in a subgroup of sporadic ccRCCs (n = 50).

	Tumor number																								
	1	9	10	12	13	15	16	18	19	20	21	23	25	26	27	28	29	30	31	32	33	34	37	38	40
**Markers**																									
D3S1560 (3p26.2)	▪	□	▪	□	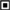	−	−	−	□	□	▪	−	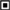	□	□	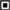	−	▪	▪	▪	−	−	−	▪	−
D3S1597 (3p25.3)	−	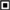	▪	−	▪	▪	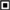	−	▪	□	−	−	▪	▪	−	▪	▪	▪	▪	▪	−	−	▪	▪	▪
*VHL gene*																									
D3S1317 (3p25.3)	−	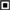	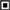	▪	▪	▪	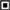	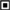	−	□	−	▪	▪	▪	−	▪	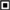	▪	−	−	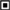	−	□	▪	▪
D3S1435 (3p25.3)	□	▪	▪	−	▪	−	−	−	▪	□	−	□	▪	−	−	−	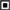	▪	▪	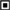	−	▪	▪	▪	−
D3S1038 (3p25.3)	−	▪	−	▪	−	▪	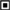	−	▪	▪	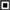	▪	▪	▪	−	▪	▪	▪	▪	□	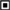	▪	□	▪	▪
D3S3611 (3p25.3)	▪	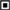	−	−	▪	▪	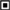	▪	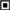	□	▪	▪	▪	−	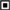	−	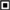	▪	−	▪	▪	−	□	−	▪
***VHL*** ** mutation**	N	N	N	Y	N	−	N	Y	N	N	Y	N	Y	Y	−	N	N	N	N	Y	Y	N	N	Y	Y

▪ = loss of heterozygosity (LOH). 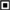 = allelic imbalance. □ = conservation of heterozygosity; − = not informative; * = allele mutated. The presence of *VHL* gene mutation (Y = yes or N = no) is also reported.

Second, the *VHL* status was also examined in 3 cases of ccpRCC and in one case of TSC-associated ccRCC since they also expressed *angptl4* mRNA. There was no *VHL* alteration in ccpRCC. An allelic imbalance was identified at D3S1317 (3p25.3) and D3S1038 (3p25.3) in TSC-associated ccRCC whereas the D3S1597 (3p25.3) and D3S1475 (3p25.3) microsatellite markers were not informative. No *VHL* mutation was detected in this case.

**Figure 2:**
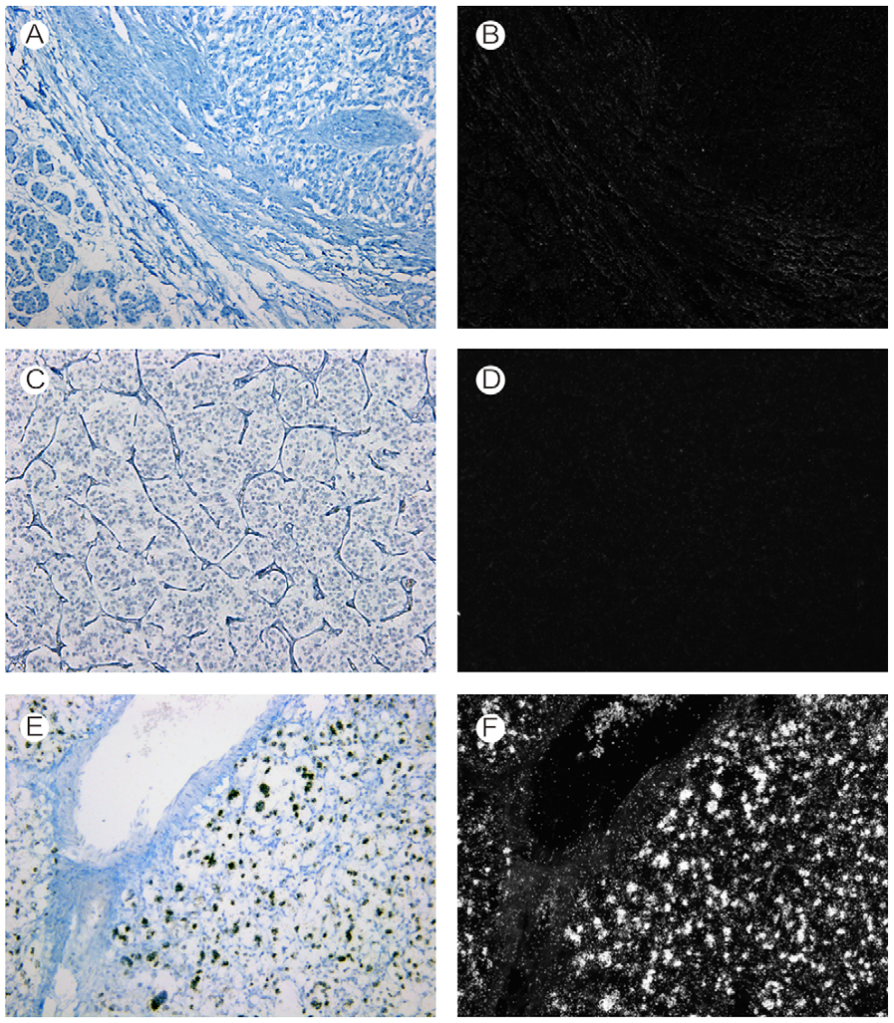
In situ hybridization analysis of *angptl4* mRNA expression in non-renal VHL disease-related tumors. Bright-field (A) and dark-field (B) views of *angptl4* mRNA expression in pancreatic endocrine tumor (up) and adjacent normal pancreas (bottom) (×10). Bright-field (C) and dark-field (D) views of *angptl4* mRNA expression in pheochromocytoma (×10). Bright-field (E) and dark-field (F) views of *angptl4* mRNA expression in hemangioblastoma (×10). Note: Absence of *angptl4* mRNA expression in pancreatic endocrine tumor (A, B), and pheochromocytoma (C, D). Strong expression of *angptl4* mRNA in hemangioblastoma (E, F).

Third, we analysed *angptl4* mRNA expression in the main non-renal tumors associated with VHL disease (ie, pancreatic endocrine and serous tumors, pheochromocytoma, and hemangioblastoma). No *angptl4* mRNA was detected by *in situ* hybridization in all pancreatic tumors analysed, independently of the *VHL* status of these tumors (Figure 2A, B, Table 2). Also, a*ngptl4* mRNA was not expressed in the 23 pheochromocytomas of this study (figure 2C, D). Among them, a missense mutation of the *VHL* gene was identified in 3 tumors (13%) which were considered as pheochromocytomas associated with a type 2c VHL disease [8], [25]. On the other hand, *angptl4* mRNA was detected in 40 (97.6%) of 41 cases of hemangioblastoma with a varying signal intensity. The negative case was associated with VHL disease. The 4 remaining cases of VHL disease-associated hemangioblastomas expressed *angptl4* mRNA moderately in 3 cases and weakly in 1 case. *angptl4* mRNA was expressed in stromal cells, which are the tumor cells in this tumor type, but not in endothelial cells (figure 1E–F). Normal pancreatic, adrenal, and cerebral tissues adjacent to tumors did not express *angptl4* mRNA. We also studied *vegf* mRNA expression in a subgroup of these non-renal VHL disease-associated tumors in order to confirm that tissue mRNA were properly preserved. High *vegf* mRNA expression was detected in all cases (data not shown).

Fourth, we sought to establish a correlation between *VHL* mutations and *angptl4* mRNA expression level in ccRCC. ccRCCs with *VHL* mutation expressed more strongly *angptl4* that ccRCCs without *VHL* mutation (*p = 0.01*, Table 3). This correlation was stronger when VHL disease-associated ccRCCs were included in this correlation study (*p = 0.004*).

## Discussion

The present study shows that *angptl4* mRNA is a powerful diagnostic marker of ccRCCs. Indeed, 100% of primary ccRCCs were *angptl4*-positive whereas 93.8% of primary non-ccRCC renal tumors were *angptl4*-negative (p = 1.5 10^−49^, Chi-square test). By comparison, the expression of CD10 for primary ccRCC is reported between 75% to 82%, and is also detected in other subtypes of RCC [11], [13]. Similarly, CA9, a hypoxia-inducible protein, and Pax-2, a homeogene strongly expressed during kidney development, are expressed in a high proportion of ccRCCs but their expression is not limited to ccRCC [10], [12]. Finally, although RCCma is the most widely used marker of RCC, it does not allow differentiation of the different histological subtypes of renal epithelial tumors [26]. *Angptl4* mRNA expression was also detected in metastases or local recurrence of ccRCC (86.7% positive in secondary ccRCCs, and 0% positive in non-ccRCC secondary RCCs). By comparison, RCCma and Pax-2 were expressed in 70% and 85% of secondary ccRCCs, respectively [26]. Further prospective studies are now needed to determine *angptl4* mRNA sensitivity, specificity and predictive values in clinical practice.

In addition to ccRCC, *angptl4* mRNA is expressed in multilocular cystic RCC (100% of cases), a putative histological variant of ccRCC [5], and in ccpRCC (100% of cases). One case (17%) of nephroblastoma also expressed *angptl4* mRNA in a focal and microcystic area formed of clear cells. Thus, *angptl4* mRNA expression seems to be characteristically associated to clear cell type of renal epithelial tumors.

Based on this observation, we further collected 39 non-renal clear cell carcinomas. Indeed, many carcinomas might present a clear cell variant and to date, there is no specific marker to differentiate metastatic clear cell carcinoma of one site from another. The specific diagnosis of origin remains a diagnosis of exclusion based on morphology and clinical/radiographic findings. We thus analysed *angptl4* mRNA level in 12 ovarian clear cell carcinomas, 20 endometrial clear cell carcinomas, and in 7 clear cell variants of both epidermoid carcinomas and adenocarcinomas of lung. No expression of *angptl4* mRNA was detected in these tumors. A recent study showed that Pax-2 more than RCCma, was an useful marker of metastatic ccRCC in a large series of tumors with clear cytoplasm [26]. Nevertheless, Pax-2 expression was noted in 42.9% of clear cell ovarian carcinomas, and no endometrial clear cell carcinoma was included in this study [26]. Therefore, our results underline the relevance of *angptl4* mRNA expression in the determination of renal origin of metastatic clear cell carcinomas.

Our laboratory has previously shown that ANGPTL4 prevents metastases by inhibiting vascular permeability as well as tumor cell motility and invasiveness in a model of xenografted melanoma B16F0 cells [16]. More recently, Padua and colleagues reported that ANGPTL4 mediated breast cancer cell metastasis to the lung in a transforming growth factor-β-dependent manner by altering endothelial integrity [19]. A hypoxic profile that included 13 genes such as *VEGF* and *ANGPTL4*, has been associated with distant metastases and might represent an independent predictor of outcomes in primary breast cancers [27]. Therefore, we have compared *angptl4* mRNA expression in regional or distant metastasis versus primary tumor of ccRCCs. The *angptl4* mRNA expression level was comparable or sometimes lower than the one observed in the primary tumor. No difference in *angptl4* mRNA expression was also noted between renal vein tumor thrombus and the adjacent primary tumor in ccRCC with renal vein extension (n = 20). Moreover *angptl4* mRNA expression level was not statistically associated with the Fuhrman nuclear grade, the tumor size, or the 2002 AJCC TNM stage groupings (*p = 0.39*, *p = 0.09*, and *p = 0.17*, respectively). Finally, the follow-up of our patients shows no difference in the occurrence of local recurrence and/or metastases (*p = 0.97*), in PFS (*p = 0.94*), and in overall survival (*p = 0.80*) according to *angptl4* mRNA expression level. Therefore, in this study, *angptl4* mRNA level has no significant prognostic value. This result is in agreement with previous expression profile studies that did not report differential expression of *angptl4* mRNA between aggressive vs. indolent ccRCCs [28], [29], [30] and ccRCCs with early *vs.* late pulmonary metastases [31]. Nevertheless, further studies are needed to confirm this point.

As *ANGPTL4* is a hypoxia-inducible gene [20], we also investigated whether *angptl4* mRNA expression in human tumors was dependent on the VHL pathway. 45 (97.8%) out of 46 sporadic ccRCCs presented a *VHL* mutation and/or a LOH at the *VHL* gene locus. These results are in agreement with previous studies which reported a *VHL* mutation in 34 to 82.4% and a LOH at *VHL* locus in up to 98% of sporadic ccRCCs [6], [32], [33]. One informative ccRCC case (case n°20, Table 5) showed no *VHL* alterations, whereas *angptl4* mRNA was highly expressed (score 3). No *VHL* alteration was identified in 3 ccpRCCs studied, as previously described [5]. An allelic imbalance was identified in 2 microsatellite markers (D3S1317 and D3S1038) located on 3p25.3 in the case of TSC-associated ccRCC.

Second, the analysis of *angptl4* mRNA expression in the main types of VHL disease-associated tumors (ie, ccRCC, pancreatic endocrine and serous tumors, pheochromocytoma, and hemangioblastoma) revealed that *angptl4* mRNA expression is restricted to ccRCCs (100% of cases) and hemangioblastomas (97.6% of cases), independently of the *VHL* status of these tumors. Our results therefore show that *VHL* gene alterations are neither necessary nor sufficient for *angptl4* mRNA expression in human tumors. Nevertheless, *VHL* mutations influence *angptl4* mRNA expression level which was higher in *VHL*-mutated ccRCCs vs. ccRCCs without *VHL* mutation (*p = 0.01*).

The present retrospective study might include some potential bias. Our cohort was intentionally chosen to increase the frequency of uncommon subtypes of renal tumors in order to constitute a comprehensive study. Thus, ccRCC represented only 54% of all cases of RCCs examined, whereas the incidence of ccRCC in daily practice is close to 75% [5], [6]. Second, as the analysis of *angptl4* mRNA expression was performed by a single centre, further multicentre validation study is needed. Third, the development of anti-ANGPTL4 antibodies for immunohistochemistry and their comparative evaluation to other immunohistochemical markers used in differential diagnosis of primary and metastatic renal tumors is required before the use of this marker in current practice in pathology laboratories. Several commercial anti-ANGPTL4 antibodies were studied by immunohistochemistry, but none of them allowed to obtain a specific staining that correlated with *angptl4* mRNA expression.

In summary, this study shows that *angptl4* is an accurate marker for primary ccRCC diagnosis but has no prognosis value for this RCC subtype. Moreover, *angptl4* mRNA expression allows to discriminate the renal origin of metastases from clear-cell carcinomas arising from various organs.

## Supporting Information

Table S1 PCR conditions and primer sets used for the sequencing analysis of the 3 exons of the *VHL* gene.
(0.03 MB DOC)

Table S2 Markers and primers used for LOH analysis at the *VHL* gene locus.
(0.03 MB DOC)
