# Supplementary material for: Determination of Angptl4 mRNA as a Diagnostic Marker of Primary and Metastatic Clear Cell Renal-Cell Carcinoma
Source: PLoS One. 2010 Apr 29;5(4):e10421. doi: 10.1371/journal.pone.0010421 (PMC2861680; doi:10.1371/journal.pone.0010421)
Supplement: Table S1 — PCR conditions and primer sets used for the sequencing analysis of the 3 exons of the VHL gene. (0.03 MB DOC) [file pone.0010421.s001.doc]

**Table S1.** PCR conditions and primer sets used for the sequencing analysis of the 3 exons of the *VHL* gene.

| Exon | **Primers (0.3 pmol of each)** | **PCR conditions** |
| --- | --- | --- |
| **Exon 1** | Primer pair 1-1  Upstream 5’AGAACTGGGACGAGGCCGA3’  Downstream 5’AGATGACCTGGGAGGGCACG3’  Primer pair 1-2  Upstream 5’GCCGAGGAGGAGATGGAG3’  Downstream 5’TTCAGACCGTGCTATCGTCCC3’ | 15 sec at 95°C,  15 sec at 65°C,  15 sec at 72°C,  40 cycles |
| **Exon 2** | Primer pair 2-1  Upstream 5’ACGAGGTTTCACCACGTTAGC3’  Downstream 5’CGTCAACATTGAGAGATGGCAC3’  Primer pair 2-2  Upstream 5’TGCTTGTCCCGATAGGTCAC3’  Downstream 5’ATTGGATAACGTGCCTGACATC3’ | 15 sec at 95°C,  15 sec at 60°C,  15 sec at 72°C,  40 cycles |
| **Exon 3** | Primer pair 3-1  Upstream 5’GACCCTAGTCTGCCACTGAGG3’  Downstream 5’ATCTCCCATCCGTTGATGTG3’  Primer pair 3-2  Upstream 5’GGAAGACCACCCAAATGTGCA3’  Downstream 5’CATCACAATGCCTAGTGAAGTCAG3’ | 15 sec at 95°,  15 sec at 60°,  15 sec at 72°C,  40 cycles |
