# Supplementary material for: Determination of Angptl4 mRNA as a Diagnostic Marker of Primary and Metastatic Clear Cell Renal-Cell Carcinoma
Source: PLoS One. 2010 Apr 29;5(4):e10421. doi: 10.1371/journal.pone.0010421 (PMC2861680; doi:10.1371/journal.pone.0010421)
Supplement: Table S2 — Markers and primers used for LOH analysis at the VHL gene locus. (0.03 MB DOC) [file pone.0010421.s002.doc]

**Table S2.** Markers and primers used for LOH analysis at the *VHL* gene locus.

| **Marker** | **Location** | **Sequence of primers** |
| --- | --- | --- |
| D3S1560 | 3p26⋅2 | Upstream 5’gCATCTACAgggggTgTCT3’  Downstream 5’gTTTCTTAggCTgATTTTCAgCACAA3’ |
| D3S1597 | 3p25⋅3 | Upstream 5’AgTACAAATACACACAAATgTCTC3’  Downstream 5’gTTTCTTgCAAATCgTTCATTgCT3’ |
| *VHL* gene | 3p25⋅3 |  |
| D3S1317 | 3p25⋅3 | Upstream 5’TACAAGTTCAGTGGAGAACC3’  Downstream 5’CCTCCAGGCCATACACAGTCA3’ |
| D3S1435 | 3p25⋅3 | Upstream 5’TggATACATTAgTATACTgAATT3’  Downstream 5’gTTTCTTTAAgACggAAgCAAggAAgg3’ |
| D3S1038 | 3p25⋅3 | Upstream 5’TCCAGTAAGAGGCTTCCTAG3’  Downstream 5’AAAGGGGTTCAGGAAACCTG3’ |
| D3S3611 | 3p25⋅3 | Upstream 5’gCTACCTCTgCTgAgCAT3’  Downstream 5’gTTTCTTTAgCAAgACTgTTgggg3’ |
